# Back to the future through the wormhole: *Caenorhabditis elegans* as a preclinical model

**DOI:** 10.1242/dmm.050333

**Published:** 2023-06-13

**Authors:** Guy A. Caldwell

**Affiliations:** ^1^ Department of Biological Sciences, The University of Alabama, Tuscaloosa, AL 35487, USA; ^2^ Department of Neurology, Center for Neurodegeneration and Experimental Therapeutics, Heersink School of Medicine, Nathan Shock Center for Research in the Basic Biology of Aging, University of Alabama at Birmingham, Birmingham, AL 35294, USA

**Keywords:** *Caenorhabditis elegans*, Preclinical, Worms, Genetics

## Abstract

On the 15th Anniversary of Disease Models & Mechanisms as a trailblazing venue for the dissemination of discoveries pertaining to human health involving model systems, we celebrate the journey of this journal, as mirrored through the evolution of research using the nematode roundworm, *Caenorhabditis elegans*. Driven by the exponential growth of genomic data, worms have advanced from a basic research tool to precise and elegant models for disease and have yielded substantive insights into numerous human disorders. A harbinger of functional genomic analysis since the inception of RNA interference screening, the directed application of *C. elegans* for identification of disease-modifying factors has revealed new pathways and therapeutic targets to accelerate translational outcomes. Together with advances in gene editing, worm models are now ushering in the era of precision medicine with characteristic expedience.

## Teaser

**Summary:** This Editorial provides a brief historical perspective on the transition of the nematode roundworm, *Caenorhabditis elegans*, from a purely basic research organism to a preclinical model for human disease.

The fine print of a standard investment agreement will inevitably include the disclaimer that “past performance may not be indicative of future results”. Inapposite to this financial tenet, science challenges us to predict future outcomes that have a basis in previously obtained results but will also be tested through empirical investigation. The match that lit the US subprime loan crisis of 2008 and spread like wildfire, ravaging financial institutions and entire economies across the globe, was borne out of speculation but fueled by a willful ignorance to facts. At that time, emerging through that haze of uncertainty was a new scientific journal – Disease Models & Mechanisms (DMM) – that challenged the risk-averse research establishment to reconsider traditional boundaries between basic and applied research. Indeed, 2008 was an age of innocence compared to today's post-COVID world of ‘alternative facts’ and artificially intelligent fakes – and rigorous science has never been more essential.

For a nascent but growing number of investigators, DMM, once established, represented more than a journal – it was a home – a venue that championed the awesome power of simple yeast cells, the elegance of lowly worms and the endlessly fruitful insights of fruit flies, in addition to a broad array of model organisms that act as means to accelerate discovery for diseases. Now, 15 years later, in the ‘post-genome’ era, DMM remains that home and continues to open its doors to research modeling disease in all systems, with even more impact on understanding, diagnosing and treating disease (Patton, 2021). Genetic screens for mutants in cell-based or invertebrate models have not been supplanted, but are now joined by voluminous datasets of human genomic variation, along with the staggering task of parsing function from these variants (Lek et al., 2022).

**Figure:**
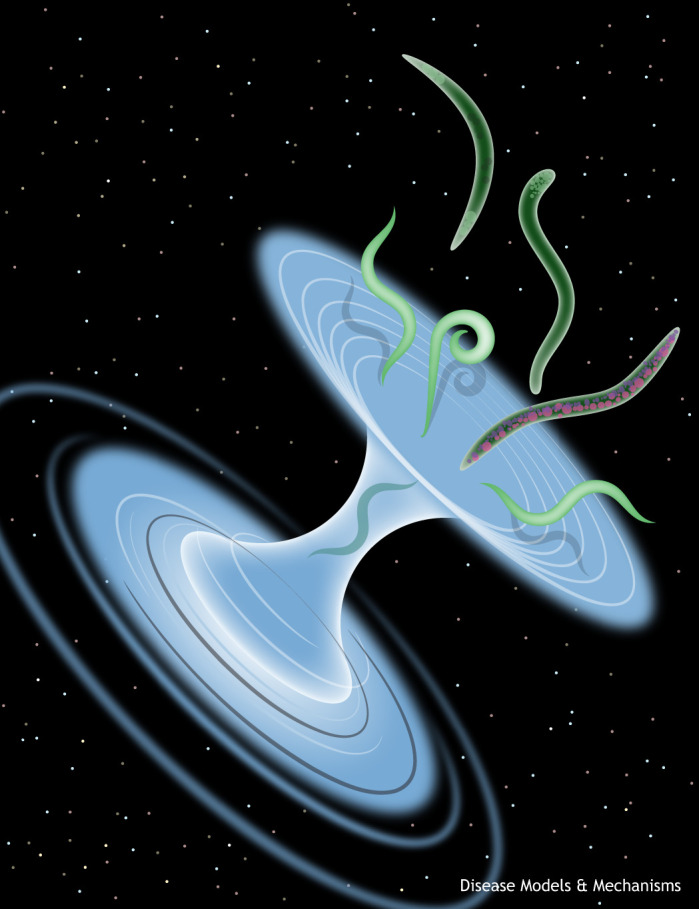


Just prior to the launch of DMM, at the 2007 International *C. elegans* Meeting, the global community of worm researchers welcomed their seminal founder, Sydney Brenner, as keynote speaker in celebration of the 2002 Nobel Prize in Physiology and Medicine, which Brenner shared with fellow worm illuminati, John Sulston and Bob Horvitz, for the co-discovery of apoptosis. Ever the iconoclast, Brenner did not dwell for a moment on past discoveries (even if he did co-discover mRNA and the genetic code) and instead shocked the standing-room-only gathering of worm-faithful researchers by boldly proclaiming, “We do not need to search for model organisms anymore; we [humans] are the new model organism”. Such statements could readily be written off as speculative fodder from most lecturers, but when an inarguably prescient sage of molecular biology like Brenner said them, the poignancy of the moment was to be respected. As documented in the pages of this journal, what followed over the next 15 years has been a steady realization of Brenner's sentiment, but facts support that yeast, worms, flies, fish and furrier friends all still have much to tell us about ourselves.

It is therefore timely that the 15th anniversary of DMM coincides with the 24th International *C. elegans* Conference, being held this June in historic Glasgow, Scotland. In anticipation of the conference, DMM showcases the versatility of the worm in an interview with Piali Sengupta discussing her work investigating sensory mechanisms (Sengupta, 2023) and in a Review from Julián Cerón on the hallmarks of cancer (Cerón, 2023). Moreover, a cursory look at the meeting program reveals multiple workshops and platform sessions devoted to the use of *C. elegans* in modeling various diseases, some involving multiple biotech companies for which it is their business to do so. As basic research rightfully remains a major driving force in the *C. elegans* field, those wading through the hundreds of conference abstracts mentioning disease might wish to trade a laboratory flask for a hip flask of a fine Scottish beverage, in preparation for a long evening. However, if a ‘State of The Worm’ address was to be given in Glasgow, it would be remiss without reference to how *C. elegans*, while still an archetypical model system for basic research, is now truly a preclinical model system as well. It is with respectful reverence to the late Dr Brenner that it must be concluded that his proclaimed end of model organism research in favor of humans as the model now appears to be premature. Whether your preference is investigations of human-derived organoids, neurons or pluripotent stem cells in culture, or genome-wide studies, it would be short sighted to deem these human sources as more pertinent to disease research than *C. elegans* or other model organisms.

Indeed, the combined knowledge that can be ascertained through use of a variety of models is invaluable in offering a more holistic view of disease biology. In repeated personal experience, our worm models have been part of concerted, collaborative, multi-organismal discovery pipelines to evaluate and prioritize genetic and/or chemical modifiers of neurodegeneration, suggesting that applying an ‘evolutionary filter’ in functional analyses provides a powerful path towards reproducible, translational outcomes (Cooper et al., 2006; Treusch et al., 2011; Tardiff et al., 2013; Knight et al., 2014; van der Welle et al., 2021; Xie et al., 2022). This collaborative approach encourages funding organizations to rethink support for individual groups to ‘do it all’ and instead provide mechanisms for multi-investigator and inter-institutional projects that facilitate more efficient and effective coordination of resources, incorporating investigators with strong expertise and proven disease models.

It is now clear that the coalescence of human clinical and genomic information with decades of experimental experience in laboratory systems enables in-depth genomic and phenotypic characterization of function (Cheng et al., 2022). The directed application of the microscopic roundworm – arguably the most well understood animal on our planet – towards discerning the functional significance of human genomic variation represents a logical progression in the arc of research, but it is also an innovative strategy to accelerate personalized or precision medicine (Mew et al., 2022).

RNA interference screening of candidate genes in *C. elegans* is a mainstay of reverse functional genomic analysis and is now complemented by the precision of CRISPR-mediated genome editing to enable functional examination of polymorphisms shared with, or analogous to, human orthologs. For rare diseases, low patient numbers or other logistical limitations are a barrier to therapeutic development; therefore, the capability to rapidly and cost-effectively model phenotypic outcomes and screen libraries of small molecule effectors in preclinical invertebrate systems can de-risk subsequent investments (Kropp et al., 2021; Ma et al., 2022). Investigations of human genomic variants of unknown significance using *C. elegans* have already served to discern the contribution (or absence) of specific polymorphisms in genes associated with ciliopathies (Lange et al., 2021, 2022), autism (Wong et al., 2019) and movement disorders (Monfrini et al., 2021; van der Welle et al., 2021; Di Rocco et al., 2022; Wang et al., 2022).

To provide more precise information and parse distinctions from within datasets, increased attention is being paid to ‘deep phenotyping’ at both the clinical and experimental level. This level of detail embraces subtilty in disease characterization and eschews traditional consensus categorizations of patients (FitzGerald et al., 2018). When assigning risk to genomic variants for different diseases, *C. elegans* prove reproducibly predictive of mammalian outcomes, especially when using endpoints that best capture translational relevance for the specific disease (Gaeta et al., 2019; Caldwell et al., 2020). Likewise, *C. elegans* is uniquely positioned as a system through which the convergence of environmental/epigenetic modulators with genetic factors implicated in disease can be simultaneously interrogated. This is exemplified in our laboratory’s recent report that revealed that dopaminergic neurodegeneration in *C. elegans* could be functionally modulated by a single protein kinase, SID-3, ortholog of human tyrosine nonreceptor kinase-2 (TNK2) (Nourse et al., 2023). Importantly, variants in the human *TNK2* gene are associated with Parkinson's disease (Farlow et al., 2016), and TNK2/SID-3 is involved in the endocytic regulation of dopamine and effectors of epigenetic gene silencing. Therefore, not only was functional annotation of disease-associated variants achieved using *C. elegans*, but this research revealed a previously undescribed *in vivo* mechanism for the epigenetic ‘tuning’ of dopamine levels, with a concomitant impact on neuronal survival (Nourse et al., 2023).

The inaugural issue of DMM contained a brief Primer, entitled ‘Traversing a wormhole to combat Parkinson's disease’, on the then relatively nascent use of *C. elegans* to model this insidious disease (Caldwell and Caldwell, 2008). In returning to that piece for the first time in over a decade, I was struck by how it ended: “Science will benefit from the efficient manner by which *C. elegans* research can contribute to the quest for translational and personalized medical breakthroughs, by boldly going where no worm has gone before”. Belated apologies to my fellow Star Trek enthusiasts aside, that statement is more pertinent than ever today. We have come to an inflection point in defining ‘model organisms’ and how the scientific community views their application. At least for worm researchers of the next generation, *C. elegans* are no longer confined to purely basic research. It is time to celebrate the diversity of final frontiers that have yet to be explored.
